# Anesthetics and long-term survival after cancer surgery—total intravenous versus volatile anesthesia: a retrospective study

**DOI:** 10.1186/s12871-019-0914-4

**Published:** 2019-12-18

**Authors:** Boohwi Hong, Sunyeul Lee, Yeojung Kim, Minhee Lee, Ann Misun Youn, Hyun Rhim, Seok-Hwan Hong, Yoon-Hee Kim, Seok-Hwa Yoon, Chaeseong Lim

**Affiliations:** 1 Department of Anesthesiology and Pain Medicine, Chungnam National University Hospital, 282 Munhwa-ro, Jung-gu, Daejeon, 35015 Republic of Korea; 2 Department of Anesthesiology and Pain Medicine, Chungnam National University College of Medicine, 266 Munhwa-ro, Jung-gu, Daejeon, 35015 Republic of Korea; 3 MediRedox (Biomedical convergence Research Center), 266 Munhwa-ro, Jung-gu, Daejeon, 35015 Republic of Korea

**Keywords:** Anesthesia, Cancer, Propofol, Surgery, Survival

## Abstract

**Background:**

Intravenous anesthesia has been reported to have a favorable effect on the prognosis of cancer patients. This study was performed to analyze data regarding the relation between anesthetics and the prognosis of cancer patients in our hospital.

**Methods:**

The medical records of patients who underwent surgical resection for gastric, lung, liver, colon, and breast cancer between January 2006 and December 2009 were reviewed. Depending on the type of anesthetic, it was divided into total intravenous anesthesia (TIVA) or volatile inhaled anesthesia (VIA) group. The 5-year overall survival outcomes were analyzed by log-rank test. Cox proportional hazards modeling was used for sensitivity.

**Results:**

The number of patients finally included in the comparison after propensity matching came to 729 in each group. The number of surviving patients at 5 years came to 660 (90.5%) in the TIVA and 673 (92.3%) in the VIA. The type of anesthetic did not affect the 5-year survival rate according to the log-rank test (*P* = 0.21). Variables associated with a significant increase in the hazard of death after multivariable analysis were male sex and metastasis at surgery.

**Conclusions:**

There were no differences in 5-year overall survival between two groups in the cancer surgery.

**Trial registration:**

Trial registration: CRIS KCT0004101. Retrospectively registered 28 June 2019.

## Background

In Korea, more than 200,000 new cancer patients are diagnosed each year and one in four deaths is due to cancer [1]. Although considerable progress has been made in chemotherapy and radiation therapy, excision of cancerous lesions remains a preferred treatment option for patients with solid tumors [2]. However, the cancer may metastasize or proliferate during surgery [3]; moreover, surgery can spread cancer cells throughout the body [4], so both doctors and patients are keenly aware of the postoperative prognosis. Cancer recurrence and metastasis are influenced by cancer propagation, patient immunity, and related factors [5].

Methods of general anesthesia for tumor resection of malignant tumors include the use of volatile anesthetics and the use of intravenous anesthetics. Several in vitro studies have investigated the use of volatile inhaled anesthetics (VIA) to increase the activation of hypoxia-inducible factor (HIF) and insulin-like growth factor, which are factors involved in tumor growth [6, 7]. There is a possibility of adverse effects on the prognosis of surgical patients. On the other hand, propofol, an intravenous anesthetic, has been reported to reduce the expression of HIF-1α and inhibit tumor growth [8].

In 2016, Wigmore et al. [9] revealed that total intravenous anesthesia (TIVA) has a favorable effect on the prognosis of cancer patients. Subsequently, many similar studies have been described in the literature. In 2017, a retrospective study showed that the use of inhalation anesthetics in 191 esophageal cancer patients had a negative effect on prognosis [10]. In a study published in 2018 regarding 1158 patients with colorectal cancer, patients who received TIVA had a better prognosis than those who received desflurane anesthesia [11]. However, other recent studies have shown that cancer prognosis is not related to the type of anesthesia [12, 13]. So far, there have been no reports that propofol-based TIVA is significantly more harmful to patient survival.

This study investigated whether 5-year overall mortality differed between patients who received propofol-based TIVA and those who received VIA during major cancer surgeries in our hospital. Based on the findings reported by Wigmore et al. [9], we hypothesized that patients who received TIVA would show a high 5-year survival rate after cancer surgery (i.e., resection for gastric, lung, liver, colon, or breast cancer), compared to patients who received VIA.

## Methods

### Setting

The study was approved by the institutional review board of Chungnam National University Hospital (approval number CNUH 2017–08-018). The requirement for informed consent was waived in view of the retrospective nature of the study. This clinical trial has been registered at Clinical Research Information Service (registration number KCT0004101).

### Participants

We reviewed the medical records of patients who underwent surgical resection for gastric, lung, liver, colon, or breast cancer from January 2006 to December 2009 in our hospital. Surgeries during the investigation period in which patients received TIVA included general and thoracic surgeries, such as thyroid, breast, colon, hepatobiliary, gastric, and lung cancer surgery. Although a high number of patients had thyroid cancer, the survival rate was sufficiently high that a comparison was not meaningful. In our hospital, thyroidectomy is rapid and it is difficult to manage intravenous catheters for affected patients; accordingly, these patients have received inhalational anesthesia for many years. Therefore, the five major cancers selected for this study were gastric, colon, liver, breast, and lung cancers.

Patients who had undergone emergency surgery, with no follow-up after surgery, patients whose medical records could not be confirmed, patients whose anesthesia was changed during surgery, and patients who died during or immediately after surgery were excluded from the study. Patients who did not fulfill any of the variables examined in the medical record were excluded. Remifentanil with 2% propofol was used via target-controlled infusion for the induction and maintenance of anesthesia in the TIVA group, while remifentanil or nitrous oxide with a volatile anesthetic agent (desflurane, sevoflurane, or isoflurane) was used for the maintenance of anesthesia in the VIA group. At the induction of anesthesia in the VIA group, propofol or etomidate was used, depending on the condition of the patient and the anesthesiologist’s preference. Because the benefits of restrictive fluid therapy were not clearly established, liberal fluid therapy was used. The type of anesthesia selected was entirely based on the anesthesiologist’s preference.

### Variables

Patient factors were age at the time of surgery, sex, body mass index (BMI), and American Society of Anesthesiologists (ASA) class. Surgical and anesthetic factors were the presence of hypertension and diabetes mellitus (DM), total anesthesia time, operation time, type of anesthesia (volatile inhalational anesthesia vs. total intravenous anesthesia), use of nitrous oxide, application of remifentanil infusion, and presence of metastasis at the time of surgery. We also investigated the patient’s total length of hospital stay. We investigated the correlations between each of the factors and 5-year survival. Patients were followed-up only with regard to the primary outcome, i.e., overall survival.

### Data sources

All data related to the surgery were obtained from the hospital statistical records. Data related to anesthesia, metastasis, and deaths were obtained from the hospital electronic medical records. If we could not find an electronic medical record of the patient’s survival at 5 years after surgery, the patient or caregiver was contacted by phone. In such instances, we briefly explained the study and received verbal consent. In addition, the contact information used at this time was not recorded on the case record sheet. If the contact information was unknown, the case was classified as a missed medical record.

### Sample size

Based on the results of a previous study [9], to achieve a power of 80% and a two-tailed type I error rate of α = 0.05, G*Power 3.1 calculations revealed that at least 495 patients were needed in each matched group. The total number of surgeries per year in our hospital is approximately 10,000; of these surgeries, approximately 600 involve surgical treatments for the five major cancers. Because the ratio between inhalation anesthesia and TIVA was approximately 2:1 during the test period, a 4-year study period was chosen. Patients who underwent surgery between 2006 and 2009 were included because 5 years had already passed at the beginning of the study. After propensity score matching, there were 729 patients in each group, which exceeded the minimum of 495 patients per group.

### Statistics

The sample consisted of all subjects during the study period. All available patients were considered. To adjust for possible selection bias and confounding factors [14], 1:1 ratio propensity score matching was performed using the MatchIt package in R [15]. The dependent variable was set as a binary response of 0 or 1, and logistic regression analysis was performed by designating the covariate (age, sex, height, weight, BMI, ASA class, hypertension, DM, anesthesia time, operation time, metastasis, transfusion) to be corrected as an independent variable. The survival rate was different for each cancer, and the numbers of anesthetic methods used were different for each cancer. Therefore, we matched for each type of cancer.

Nearest neighbor matching was performed, which matches the absolute differences of the estimated propensity scores of all subjects in both groups from the smallest to the largest difference. Absolute standardized difference (ASD) was calculated to validate the suitability of propensity score matching balance diagnostics between the two groups, with ASD < 0.1 for the covariate indicating that the two groups were sufficiently balanced.

After validating the balance of the matched data, the normality of continuous data was assessed using the Shapiro–Wilk test. If normality was satisfied, comparisons between groups were performed by independent t tests, with the results expressed as means ± standard deviations. If normality was not satisfied, groups were compared using the Mann–Whitney U test, with the results expressed as medians (interquartile ranges). Categorical data were compared using the chi-squared test or Fisher’s exact test, as appropriate, with the results expressed as numbers (%).

Survival outcomes were analyzed by the log-rank test and expressed by the Kaplan–Meier plot. Cox proportional hazards modeling was used for univariate and multivariable analysis of demographic and clinical variables influencing the survival outcomes. The cut points of the continuous variables were obtained using the maxstst package; survival analysis was performed by separating the patients into two categories based on the following cut points: age, 65 years; height, 165 cm; weight, 57 kg; BMI, 19.7; and anesthesia time, 210 min. Only the meaningful variables (*P* < 0.2) from univariate analysis were included in multivariable analysis. Akaike’s Information Criterion was considered for final model selection by backward elimination. Associations with *P* < 0.05 were considered statistically significant. All Data were analyzed using R software version 3.5.2 (R Project for Statistical Computing, Vienna, Austria).

## Results

**Fig. 1:**
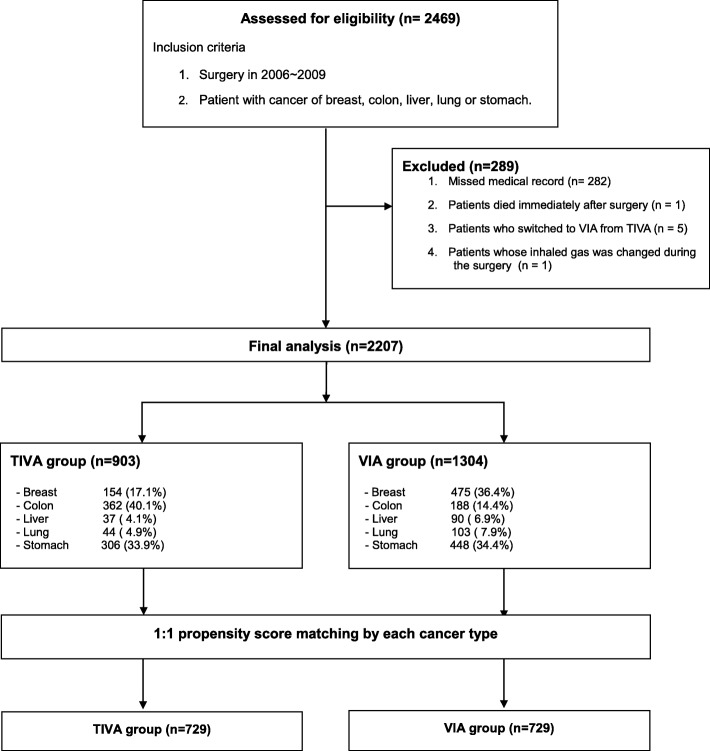
Flow diagram. TIVA = total intravenous anesthesia; VIA = volatile inhaled anesthesia

We reviewed the following items in the anesthesia and operation records of patients who underwent surgery. From January 2006 to December 2009, 2496 patients underwent resection of five major malignant tumors. After exclusion of 289 patients according to the exclusion criteria, the analysis included a total of 2207 patients (Fig. 1). All patient information is shown in Table 1. Anesthesia was maintained by inhalation anesthesia in 1304 patients and TIVA in 903 patients undergoing surgery. The numbers of patients finally included in the comparison after propensity score matching were 729 in each group.

**Table 1:** Data for Patients Overall and Matched Patients after Propensity Scoring

	Overall Patients				Matched Patients			
Variables	TIVA (*n* = 903)	VIA (*n* = 1304)	ASD	*P*	TIVA (*n* = 729)	VIA (*n* = 729)	ASD	*P*
Age, yr	58.0 [49.0;67.0]	57.0 [48.0;67.0]	0.098	0.018	58.0 [49.0;67.0]	57.0 [48.0;67.0]	0.003	0.861
Sex			0.233	< 0.001			0.019	0.753
Female	404 (44.7%)	734 (56.3%)			337 (46.2%)	344 (47.2%)		
Male	499 (55.3%)	570 (43.7%)			392 (53.8%)	385 (52.8%)		
Height, cm	161.0 [154.0;166.0]	159.0 [154.0;165.0]	0.118	0.007	161.0 [154.0;166.0]	161.0 [155.0;166.0]	0.015	0.610
Weight, kg	60.0 [54.0;67.0]	60.0 [54.0;67.0]	0.002	0.368	60.0 [54.0;67.0]	60.0 [53.0;67.0]	0.023	0.527
BMI, kg m^−2^	23.6 [21.8;25.8]	23.8 [21.8;26.1]	0.062	0.210	23.6 [21.7;25.8]	23.5 [21.5;25.8]	0.039	0.577
ASA class			0.098	0.067			0.053	0.605
I	399 (44.2%)	633 (48.5%)			311 (42.7%)	310 (42.5%)		
II	503 (55.7%)	671 (51.5%)			417 (57.2%)	419 (57.5%)		
III	1 (0.1%)	0 (0.0%)			1 (0.1%)	0 (0.0%)		
Hypertension			0.008	0.894			< 0.001	1.000
Yes	233 (25.8%)	341 (26.2%)			188 (25.8%)	188 (25.8%)		
No	670 (74.2%)	963 (73.8%)			541 (74.2%)	541 (74.2%)		
DM			0.057	0.204			0.032	0.593
Yes	121 (13.4%)	150 (11.5%)			95 (13.0%)	103 (14.1%)		
No	782 (86.6%)	1154 (88.5%)			634 (87.0%)	626 (85.9%)		
Anesthesia time, min	230.0 [185.0;285.0]	210.0 [170.0;260.0]	0.166	< 0.001	215.0 [180.0;260.0]	220.0 [180.0;265.0]	0.017	0.696
Operation time, min	190.0 [150.0;240.0]	175.0 [135.0;220.0]	0.146	< 0.001	180.0 [149.0;220.0]	180.0 [149.0;225.0]	0.012	0.787
Remifentanil infusion				< 0.001				< 0.001
Yes	902 (99.9%)	701 (53.8%)			728 (99.9%)	395 (54.2%)		
No	1 (0.1%)	603 (46.2%)			1 (0.1%)	334 (45.8%)		
Gas type				< 0.001				< 0.001
Des	0 (0.0%)	345 (26.5%)			0 (0.0%)	193 (26.5%)		
Iso	0 (0.0%)	31 (2.4%)			0 (0.0%)	25 (3.5%)		
Sevo	0 (0.0%)	927 (71.1%)			0 (0.0%)	511 (70%)		
Nitrous oxide				< 0.001				< 0.001
Yes	1 (0.1%)	550 (42.2%)			1 (0.1%)	302 (41.4%)		
No	902 (99.9%)	754 (57.8%)			728 (99.9%)	427 (58.6%)		
Metastasis at surgery			0.006	0.947			0.014	0.855
Yes	68 (7.5%)	96 (7.4%)			64 (8.8%)	67 (9.2%)		
No	835 (92.5%)	1208 (92.6%)			665 (91.2%)	662 (90.8%)		
Transfusion			0.056	0.252			0.038	0.626
Yes	19 (2.1%)	39 (3.0%)			10 (1.4%)	7 (1.0%)		
No	884 (97.9%)	1265 (97.0%)			719 (98.6%)	722 (99.0%)		
Hospital stay, day	13.0 [11.0;18.0]	13.0 [10.0;18.0]	0.014	0.238	13.0 [11.0;18.0]	13.0 [10.0;18.0]	0.023	0.355
Survival, month	60.0 [44.0;60.0]	60.0 [45.0;60.0]		0.945	60.0 [40.0;60.0]	60.0 [47.0;60.0]		0.523
5 years survival				0.291				0.262
Yes	829 (91.8%)	1214 (93.1%)			660 (90.5%)	673 (92.3%)		
No	74 (8.2%)	90 (6.9%)			69 (9.5%)	56 (7.7%)		
Cancer type				< 0.001				1.000
Breast	154 (17.1%)	475 (36.4%)			154 (21.1%)	154 (21.1%)		
Colon	362 (40.1%)	188 (14.4%)			188 (25.8%)	188 (25.8%)		
Liver	37 (4.1%)	90 (6.9%)			37 (5.1%)	37 (5.1%)		
Lung	44 (4.9%)	103 (7.9%)			44 (6.0%)	44 (6.0%)		
Stomach	306 (33.9%)	448 (34.4%)			306 (42.0%)	306 (42.0%)		

Number (%): chi-square test, median [interquartile range]: Mann–Whitney U test

*ASD* Absolute standardized mean difference, *BMI* Body mass index, *ASA* American Society of Anesthesiologists, *TIVA* Total intravenous anesthesia, *VIA* Volatile inhalational anesthesia, *DM* Diabetes mellitus, *Des* Desflurane, *Iso* Isoflurane, *Sevo* Sevoflurane

### Anesthesia

In the TIVA group, all patients used propofol, and all patients were treated with remifentanil, except one patient treated with alfentanil. One patient in the TIVA group was treated with nitrous oxide, which was administered within 5 min after induction of anesthesia because the anesthesia machine was set up to automatically administer nitrous oxide when the fraction of inspired oxygen was reduced. Among the 1304 patients in the VIA group, remifentanil was administered to 701 and nitrous oxide was administered to 550; fentanyl was continuously or intermittently administered to the remaining 53 patients. No patients received epidural pain control or regional block.

### Five-year survival: TIVA vs. VIA

**Fig. 2:**
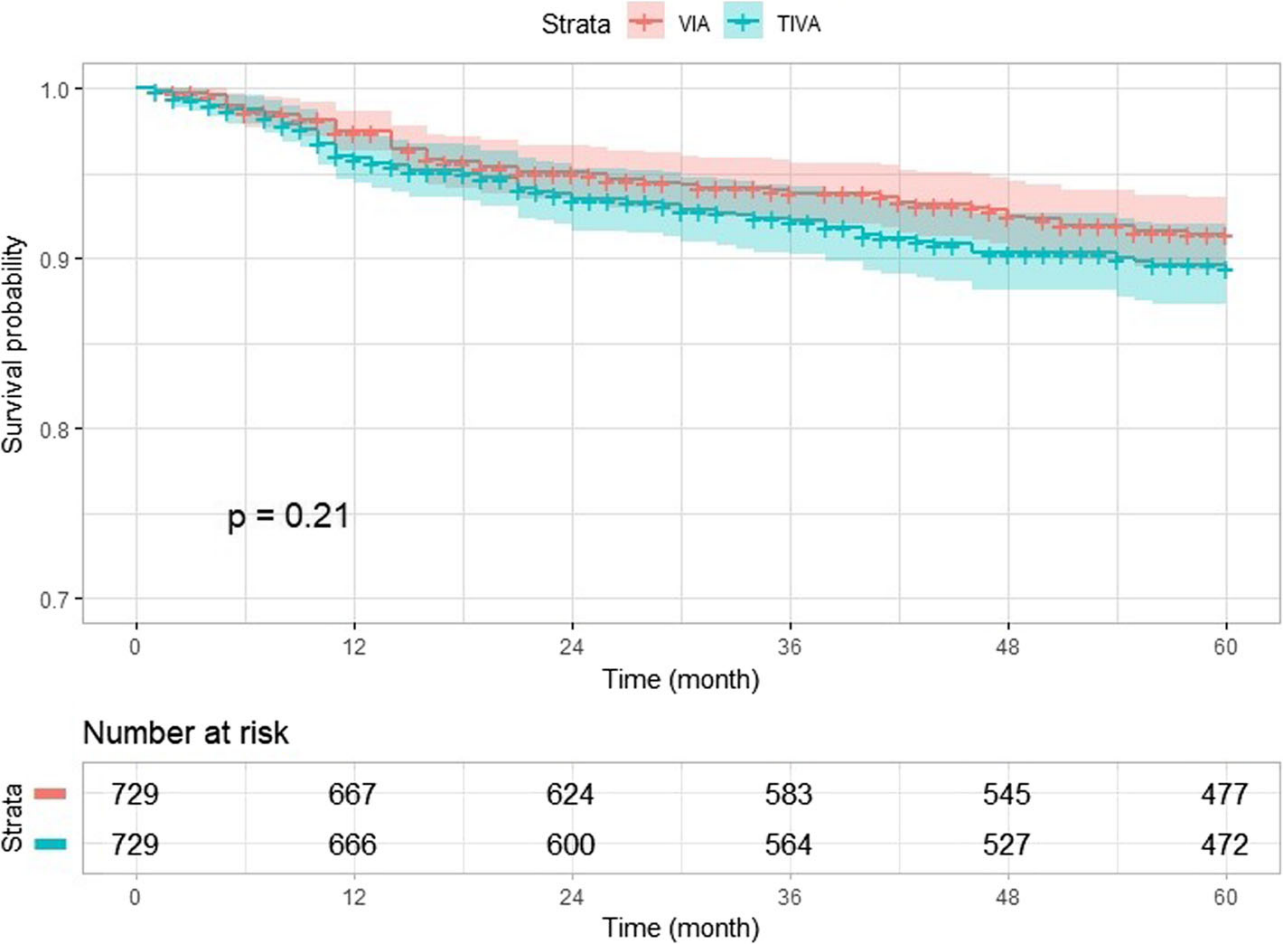
Comparison of survival rate by Kaplan–Meier survival curves after propensity matching. VIA = volatile inhaled anesthesia group; TIVA = total intravenous anesthesia group

The numbers of surviving patients at 5 years were 829/903 (91.8%) in the TIVA group and 1214/1304 (93.1%) in the VIA group; after propensity score matching, these numbers were 660/729 (90.5%) and 673/729 (92.3%), respectively. The type of anesthetic did not affect the 5-year survival rate, according to log-rank analysis, as shown in the Kaplan–Meier plot in Fig. 2 (*P* = 0.21). The type of anesthetic showed no correlation with survival, even in univariate analysis (HR = 1.26, CI = 0.88 to 1.79, *P* = 0.21).

### Sensitivity analysis: multivariable cox regression analysis

The hazard ratios of the groups in the univariate model for the propensity score-matched groups are shown in Table 2. Male sex, high BMI, long anesthesia time, and metastasis affected risk of death in the univariate model. The hazard ratios of the groups in the multivariable model for the propensity score-matched groups are shown in Table 3. Variables associated with significant increases in the risk of death after multivariable analysis were male sex and the presence of metastasis at surgery. Only five variables were included in multivariable analysis, based on the selection of meaningful variables (*P* < 0.2) from univariate analysis.

**Table 2:** Hazard Ratios by Univariate Model

	HR	95% Cl	*P*-value
Anesthesia type: TIVA vs VIA	1.255	0.882 to 1.785	0.206
Age, yr: > 65 vs ≤ 65	1.000	0.988 to 1.019	0.616
Sex: male vs female	1.602	0.990 to 1.034	0.011*
Height, cm: > 166 vs ≤ 166	1.012	0.990 to 1.004	0.283
Weight, kg: > 57 vs ≤ 57	0.932	0.883 to 0.983	0.117
BMI, kg m^−2^: > 19.7 vs ≤ 19.7	0.932	0.883 to 0.983	0.010*
ASA class: II vs I	1.248	0.870 to 1.790	0.228
Hypertension: no vs yes	1.200	0.788 to 1.828	0.394
DM: no vs yes	0.903	0.548 to 1.489	0.690
Anesthesia time, min: > 210 vs ≤ 210	1.003	1.001 to 1.005	0.002**
Metastasis: no vs yes	0.123	0.085 to 0.179	< 0.001**
Transfusion: no vs yes	0.684	0.169 to 2.769	0.595

*BMI* Body mass index, *ASA* American Society of Anesthesiologists, *HR* Hazard ratio, *TIVA* Total intravenous anesthesia, *VIA* Volatile inhaled anesthesia, *DM* Diabetes mellitus; *P < 0.05; **P < 0.01

**Table 3:** Hazard Ratios by Multivariable Analysis

	Cancer type included			Cancer type excluded		
	HR	95% Cl	*P*-value	HR	95% Cl	*P*-value
Sex: male vs female	1.031	0.625 to 1.700	0.905	1.731	1.078 to 2.781	0.023*
Weight, kg: > 57 vs ≤ 57	0.990	0.956 to 1.026	0.591	0.980	0.946 to 1.016	0.275
BMI, kg m^−2^: > 19.7 vs ≤ 19.7	0.983	0.889 to 1.087	0.743	1.015	0.917 to 1.122	0.777
Anesthesia time, min: > 210 vs ≤ 210	1.001	0.998 to 1.003	0.573	1.000	0.998 to 1.002	0.911
Metastasis: no vs yes	0.119	0.078 to 0.180	< 0.001**	0.132	0.088 to 0.199	< 0.001**
Cancer type						
Breast (reference)						
Colon	2.594	0.917 to 7.341	0.072			
Liver	8.168	2.556 to 26.104	< 0.001**			
Lung	7.235	2.295 to 22.810	< 0.001**			
Stomach	6.576	2.533 to 17.069	< 0.001**			

Only variables with a significance level of *P* < 0.2 in univariable analysis were included in the multivariable model. *BMI* Body mass index, *ASA* American Society of Anesthesiologists, *HR* Hazard ratio, *TIVA* Total intravenous anesthesia, DM Diabetes mellitus; *P < 0.05; **P < 0.01

### Survival rates of each cancer

**Fig. 3:**
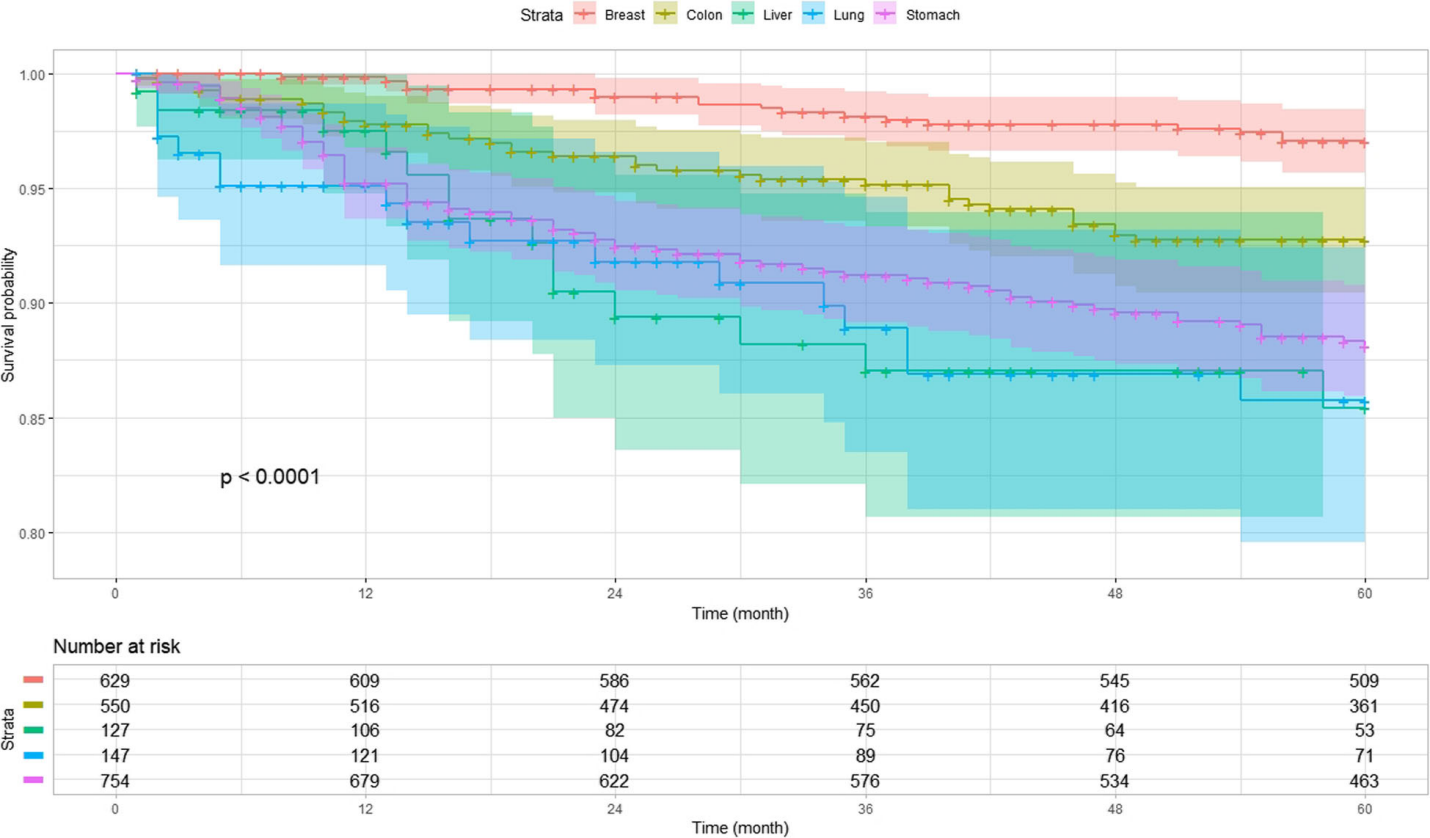
Kaplan–Meier survival curves grouped by cancer type

**Fig. 4:**
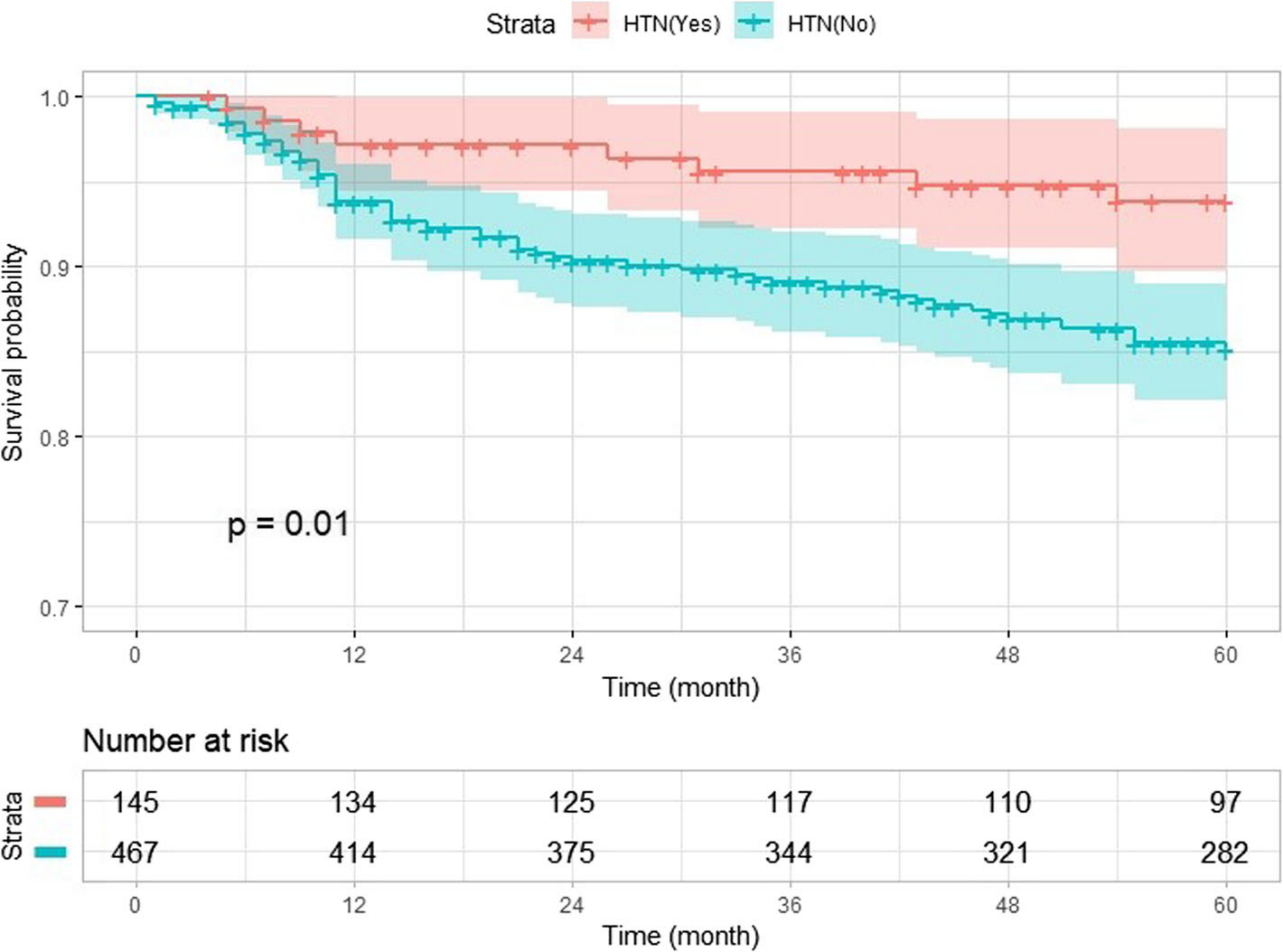
Kaplan–Meier survival curves according to hypertension after propensity matching in stomach cancer patients. HTN = hypertension history

Survival was highest in patients with breast cancer, followed by patients with colon and stomach cancers; similar mortalities were observed in patients with lung and liver cancers (Fig. 3). We divided the patients based on the types of cancer and analyzed whether the factors from multivariable analysis influenced survival differently among the groups. The results of this subgroup analysis were similar to those of all cancers combined, with the exception of stomach cancer patients without hypertension, who had a low survival rate according to the log-rank test; this is shown in the Kaplan–Meier plot in Fig. 4 (*P* = 0.01).

## Discussion

In this study, there was no effect of TIVA or VIA on the survival rate of the overall population of patients undergoing surgery for the five major types of cancer. There was no significant association between the type of anesthetic used and prognosis following cancer surgery.

Each anesthetic has a unique effect on immune regulation and cancer growth factor production [16–19]. It has been reported that propofol exhibits better immunomodulatory properties than volatile anesthetics [20–22]. Some studies have shown that survival rates after cancer surgery are better for patients who receive TIVA than for those who receive VIA [9, 10, 23, 24]. After matching, postoperative survival was investigated in 1158 patients with colon cancer [11]; the propofol-treated group had better survival (189 deaths, 32.6%, in the desflurane group vs. 87, 15.0%, in the propofol group). A recent study showed that propofol was associated with better survival after surgery in 670 patients with hepatocellular carcinoma [25]. For patients with breast cancer, propofol may reduce the relapse rate within 5 years, but a study of patients in the Korea Cancer Center showed no difference in 5-year survival based on the type of anesthetic used during surgery [26]. A comparison of 3532 patients with breast cancer at Seoul National University Hospital revealed no differences in recurrence-free survival and overall survival, based on the type of anesthetic used during surgery [13]. An analysis of 1794 patients with gastric cancer demonstrated that TIVA was associated with better survival after surgery [27]. Depending on the time of gastric cancer resection surgery, some patients had a long-term survival of 80–90 months. Another study of 1538 patients with gastric cancer found that propofol-based TIVA had no significant effect on 1-year overall survival or cancer-related mortality after surgery, but this could have been related to the short 1-year study period [12]. Finally, a study of 392 patients with non-small cell carcinoma showed no benefit for long-term prognosis when TIVA was used during surgery [28].

Thus far, the findings have differed among studies depending on the type of cancer, the research institute involved, the duration of the investigation, and whether overall survival or recurrence-free survival is assessed. However, there have been no reports that propofol-based TIVA is significantly more harmful to patient survival. Although it did not include the most recent reports, a meta-analysis of 21,000 patients showed that both recurrence-free survival and overall survival rates were higher in the TIVA group than in the volatile anesthesia group [29]. Despite these data, one survey revealed that most anesthesiologists preferred inhalation anesthesia [30]. As many as 43% of respondents presumed that TIVA could reduce cancer recurrence; however, only 29% of them used TIVA for cancer surgery. Factors affecting cancer prognosis are very diverse and complex; therefore, they may not differ simply because of the anesthetic used. In our hospital, regardless of whether the surgery involves cancer treatment, most anesthesiologists use sevoflurane or desflurane for general anesthesia. Notably, the proportion of patients who received TIVA for general anesthesia in 2018 at Chungnam National University Hospital was 1575 of 12,659 (12%). This is likely because the benefits of the TIVA are not yet clear and a syringe infuser is not available.

There have been several reports that neither TIVA nor volatile anesthesia affected the prognosis of cancer patients [13, 28, 31], and the present study was consistent with these results. In this study, hypertension was shown to be associated with 5-year survival only in gastric cancer patients on univariate analysis. As the effect of medication taken daily by hypertensive patients has not been investigated, it will be difficult to estimate accurately the mechanism underlying this observation. As observed in patients with gastric cancer in this study, hypertension may provide a survival advantage, as indicated in a study of women with ovarian cancer [32]. New research from epidemiologists at Roswell Park Cancer Institute provided evidence that hypertension and diabetes as well as the use of medications to treat these common conditions may influence the survival of ovarian cancer patients. Hypertension was reported to be associated with lower risk of disease progression among patients with endometrioid tumors (*n* = 339, HR = 0.54; 95% CI = 0.35 to 0.84). In Korea, hypertension is treated indiscriminately by combination therapy with aspirin or statins, which may be another explanation for these observations. Aspirin use may have only a small effect on gastric carcinoma [33]. One meta-analysis [34] showed that statins were inversely related to the risk of gastric cancer (RR = 0.56; 95% CI = 0.35 to 0.90). Thus far, there is no clear explanation for the good prognosis we observed in patients who take medications for hypertension control, especially among patients with stomach cancer. To explain this observation, further studies are required to determine which medications were taken daily by patients with hypertension who underwent surgery for stomach cancer.

This study had some limitations, primarily due to its retrospective nature. The size of the study population was also small, although this was partially addressed by propensity score matching. Furthermore, overall survival was used as the primary outcome. Thus, we did not distinguish among deaths from cancer recurrences, deaths from other diseases, or sudden accidents. However, considering the very long average life span of Koreans [35], we considered this unlikely to be a problem. This use of overall survival may be why multivariable Cox regression analysis showed that age was not a significant covariate. The final limitation was that no special fluid therapy, mechanical ventilation, or postoperative management was included.

## Conclusions

There were no differences in 5-year overall survival between the TIVA and VIA groups in patients who underwent major cancer surgeries in our hospital. Therefore, we cannot conclude that propofol-based TIVA is more suitable than VIA for use in cancer surgery. Unexpectedly, patients with stomach cancer showed better survival when they had hypertension than when they did not have hypertension. To increase the objectivity of these results, further studies with a larger number of patients are needed.

## Data Availability

The raw data of the current study are available from the corresponding author on request.

## Abbreviations

ASA: American society of anesthesiologists
ASD: Absolute standardized difference
BMI: Body mass index
CRIS: Clinical research information service
DM: Diabetes mellitus
HIF: Hypoxia-inducible factors
PSM: Propensity score matching
TIVA: Total intravenous anesthesia
VIA: Volatile inhaled anesthesia
